# An unusual case of ST-segment elevation myocardial infarction following a late bare-metal stent fracture in a native coronary artery: a case report

**DOI:** 10.1186/1752-1947-3-9296

**Published:** 2009-11-24

**Authors:** Giovanni Minardi, Paolo G Pino, Marco Stefano Nazzaro, Herribert Pavaci, Martina Sordi, Cesare Greco, Carlo Gaudio

**Affiliations:** 1 Department of Cardiology and Cardiovascular Surgery, Azienda Ospedaliera San Camillo-Forlanini, Rome, Italy; 2 Department of Heart and Great Vessels Attilio Reale, Second Division of Cardiology, "Sapienza", University of Rome, Rome, Italy

## Abstract

**Introduction:**

A bare-metal stent fracture as a cause of acute coronary thrombosis and consequently of acute coronary syndrome is a rare clinical event that, to the best of our knowledge, has previously not been reported. A stent fracture is a rare complication arising from percutaneous coronary intervention.

**Case presentation:**

We present, to the best of our knowledge, the first documented case of ST-segment elevation myocardial infarction in a patient following a late bare-metal stent fracture and thrombosis in a native coronary artery. The patient, a 51-year-old Caucasian man, was treated successfully with primary percutaneous coronary intervention and a new stent implantation.

**Conclusion:**

A coronary stent fracture is a rare complication that has been described in venous bypass grafts deploying either a drug-eluting stent or a bare-metal stent. Stent fractures rarely occur in coronary arteries. In light of the non-specific presentation of stent fracture, it is also an easily missed complication. Patients may present with a non-specific symptom of angina. The angina could either be stable or unstable as a result of restenosis or in-stent thrombosis, or both. Our case demonstrates the most severe consequences of a bare-metal stent fracture (sudden coronary thrombosis and subsequent myocardial infarction) in a native coronary artery. It was diagnosed angiographically and treated early and effectively.

## Introduction

A bare-metal stent (BMS) fracture as a cause of acute coronary thrombosis and consequently of acute coronary syndrome (ACS) is a rare clinical event that, to the best of our knowledge, has previously not been reported.

A stent fracture is a rare complication of percutaneous coronary intervention (PCI). Drug-eluting stent (DES) fractures have an estimated incidence of 2.7% [1], and a BMS fracture in a saphenous vein graft has recently been described [2]. A late BMS fracture has also been reported, which was detected by 64-slice multidetector computed tomography (MDCT) [3].

## Case presentation

A 51-year-old Caucasian man who smoked and was afflicted with dyslipidemia presented at our emergency department complaining of typical angina and shortness of breath. He had a family history of coronary artery disease. He had been successfully treated with coronary angioplasty 12 years before presentation. A 4.0/16.0 mm AVE Micro stent (AVE Inc., Santa Rosa, CA, USA) was deployed in the proximal left anterior descending artery (LAD). The stent was postdilated with a 4.0-mm high dilatation force balloon (High Energy) at 14 atmospheres with good angiographic results. The patient was on therapy with aspirin, a statin, omega-3 fatty acids and a β-blocker and had remained asymptomatic up to his admission.

**Figure 1:**
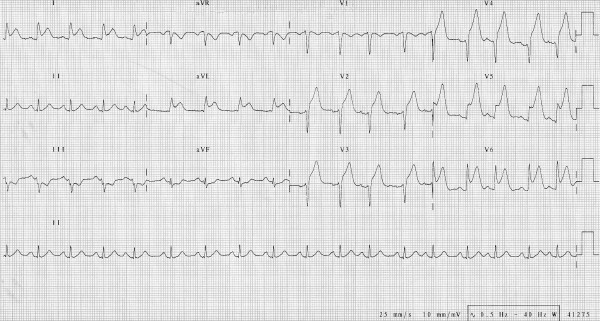
**A 12-lead electrocardiography on admission indicating an anterior ST segment myocardial infarction**.

An electrocardiogram showed a marked ST-segment elevation in leads V2-V6, I and aVL, suggesting an extended anterior acute myocardial infarction (Figure 1).

**Figure 2:**
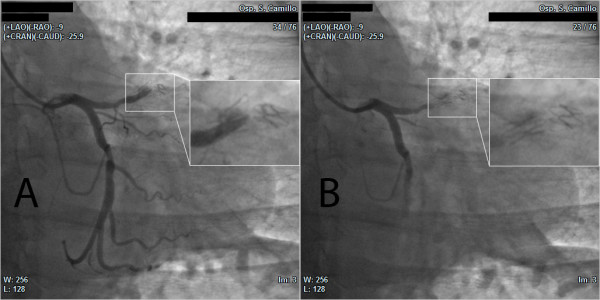
**Coronary angiogram showing the proximal occlusion of the left anterior descending artery (A) and stent fracture at a higher magnification (B)**.

Emergency coronary angiography revealed total occlusion of the LAD with complete fracture of the mid portion of the stent (Figure 2 and Additional file 1). The circumflex artery had a non-critical plaque in the mid portion (50%) and the right coronary artery was normal.

**Figure 3:**
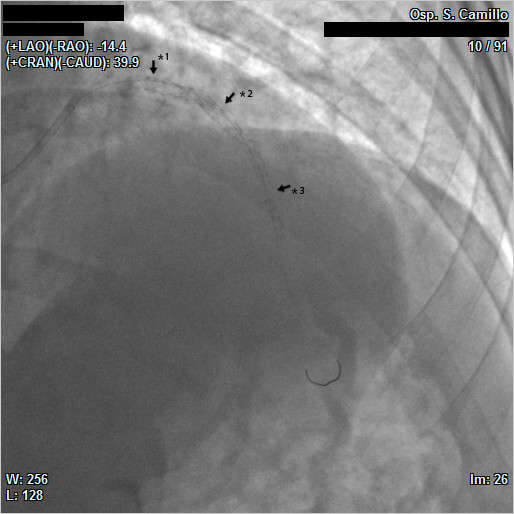
Final angiogram of the left anterior descending artery showing three Cypher stents placed in the fractured area: (*1) proximal (3.5 × 8 mm), (*2) middle (3.0 × 33 mm) and (*3) distal (2.75 × 28 mm).

A percutaneous coronary intervention (PIC) was performed. After predilation, two Cypher stents (Cordis, Miami Lakes, FL, USA) 3.0 × 33 mm in the middle part and 3.5 × 8 mm proximally, respectively, were superposed on the previous stent in the fractured occlusion area. Subsequently, a third Cypher stent 2.75 × 28 mm in the mid-LAD was deployed (Figure 3 and Additional file 2, Additional file 3 and Additional file 4).

**Figure 4:**
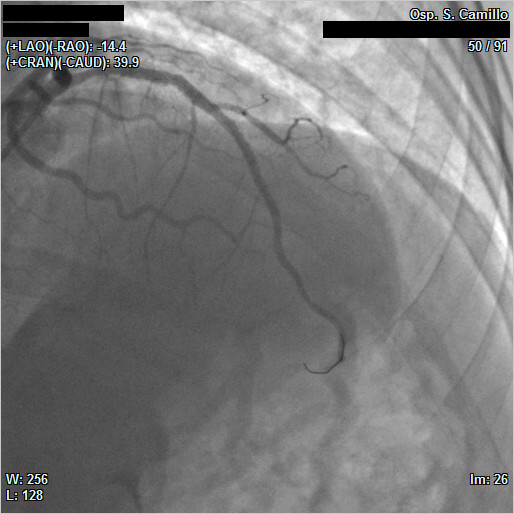
**Final angiographic result: complete opacification of the left anterior descending artery**.

Overlapped segments with extensor balloon (3.5 × 20 mm) postdilation were performed. The affected coronary artery was successfully reperfused (final TIMI 3 flow) (Figure 4 and Additional file 5). The peak troponin T level was 82.89 ng/ml, myoglobin was greater than 1000 ng/ml, and creatine kinase-MB level was 217.30 ng/ml.

The patient was admitted to the coronary care unit and treated with double antiplatelet therapy (aspirin and clopidogrel) as well as a β-blocker, ACE-inhibitor, statin and diuretic. The patient's later course was uneventful, as cardiac biomarkers normalized.

## Conclusion

Stent fractures have been reported in vascular settings such as in the iliac artery [4] and the subclavian artery [5]. The mechanism of fracture has long been considered to be excessive mechanical stress due to extreme contraction and/or flexion of the vessel.

Coronary stent fracture is a rare complication and has been described in venous bypass grafts, deploying either DES or BMS [2]. In venous grafts, mechanical stress can be very high and may lead to coronary stent fracture. In addition, aggressive postdilation of a deployed stent may also lead to stent fracture.

Stent fractures occur very rarely in native coronary arteries. Around 2.7% of patients treated with DES have experienced a stent fracture [1,6-9] and BMS can also break. Recently, BMS fractures have been detected in a 64-slice MDCT [3,10]. The potential risk of stent fracture must be considered when dealing with stent-to-vessel undersizing with subsequent aggressive postdilation. If the struts of the stent are compromised during this process, then the risk of stent fracture is significantly increased [5]. Due to higher radial forces, longer stents can be more prone to fracture than shorter ones. Stent fractures have also been observed with overlapping stents.

Because of the non-specific presentation of a stent fracture, it is a complication that can easily be missed. Patients may present with non-specific symptoms of angina which could be attributed to either stable or unstable angina as a result of restenosis or in-stent thrombosis, or both. In order to ensure proper recognition and treatment of this entity, physicians must be aware of its existence and the possibility of its occurrence under certain circumstances.

The incidence of these events could be underestimated because the widespread and slow tissue overgrowth inside the stent may mask the fracture. However, it is believed that not all stent fractures are associated with clinical sequelae so it is difficult to recognize them without repeat angiography. Stent thrombosis may result in death prior to hospitalization so it is possible that some cases of sudden death following DES implantation may result from unrecognized stent fracture.

This case demonstrates the most severe presentation of a BMS fracture, with sudden coronary thrombosis and subsequent myocardial infarction, which was diagnosed angiographically and treated early and effectively.

It is worth noting that the patient had no significant stent fracture predictors such as stent length (it was a relatively short stent), aggressive expansion and location (saphenous vein graft or right coronary artery).

This case describes one type of presentation of a stent fracture and how important it is to recognize a stent fracture, even if it is a rare occurrence. It is also important to note this in the management of patients who present with chest pain many years after percutaneous coronary intervention (PCI).

## Abbreviations

ACS: acute coronary syndrome; BMS: bare-metal stent; DES: drug-eluting stent; LAD: left anterior descending artery; MB: muscle brain; MDCT: multidetector computed tomography; PCI: percutaneous coronary intervention.

## Competing interests

The authors declare that they have no competing interests.

## Authors' contributions

GM, PGP and CGa made substantial contributions in conceptualizing and designing this study. MSN and CGr performed the PCI. GM, HP and MS were involved in drafting the manuscript and revising it critically. All authors have given final approval of the version to be published.

## Consent

Written consent was obtained from the patient for publication of this case report and any accompanying images. A copy of the written consent is available for review by the Editor-in-Chief of this journal.

## Supplementary Material

Additional file 1 Total occlusion of the LAD with complete fracture of mid portion of the stent. Click here for file

Additional file 2 Cypher stent (3.0 × 33 mm) is superposed on the previous stent in the fractured occlusion area. Click here for file

Additional file 3 Cypher stent (3.5 × 8 mm) in the proximal part is superposed on the previous stent in the fractured occlusion area. Click here for file

Additional file 4 Third stent, Cypher (2.75 × 28 mm) in the mid-LAD is deployed. Click here for file

Additional file 5 Final angiogram showing successful reperfused culprit coronary artery. Click here for file
